# Supplementary material for: UV-B Radiation Exhibited Tissue-Specific Regulation of Isoflavone Biosynthesis in Soybean Cell Suspension Cultures
Source: Foods. 2024 Jul 28;13(15):2385. doi: 10.3390/foods13152385 (PMC11312073; doi:10.3390/foods13152385)
Supplement: Supplementary file 1 [file foods-13-02385-s001.zip › foods-3116472-supplementary.pdf]

Supplementary material for

UV-B Radiation Exhibited Tissue-Specific Regulation of Isoflavone Biosynthesis in Soybean Cell Suspension Cultures

Mian Wang [1], Yiting Wang [1], Muhammad Bilal [1], Chong Xie [1,2], Pei Wang [1,2], Xin Rui [1] and Runqiang Yang [1,2,*]

[1] College of Food Science and Technology, Whole Grain Food Engineering Research Center, Nanjing Agricultural University, Nanjing 210095, China; 2018108018@njau.edu.cn (M.W.); 2023908007@stu.njau.edu.cn (Y.W.); 2022108181@stu.njau.edu.cn (M.B.); xiechong@njau.edu.cn (C.X.); wangpei@njau.edu.cn (P.W.); ruix@njau.edu.cn (X.R.)
[2] Sanya Institute of Nanjing Agricultural University, Sanya 572024, China
* Correspondence: yangrq@njau.edu.cn; Tel./Fax: +86-025-84396293

Supplementary Materials

1. Results

1.1. Typical HPLC chromatograms of nine isoflavone monomers in soybean hypocotyl and cotyledon cell suspension cultures

Typical HPLC chromatograms of nine isoflavone monomers were shown in Figure S1. Nine primary isoflavones were identified in Figure S1a. As shown in Figure S1b, daidzin, genistin, malonyldaidzin, malonylglycitin and malonylgenistin were the main isoflavones in soybean hypocotyl cell suspension cultures. Interestingly, glycitin, malonyldaidzin, malonylgenistin and daidzein were detected in soybean cotyledon cell suspension cultures (Figure S1c). Additionally, daidzin, malonylglycitin and genistein were detected in UV-B group.

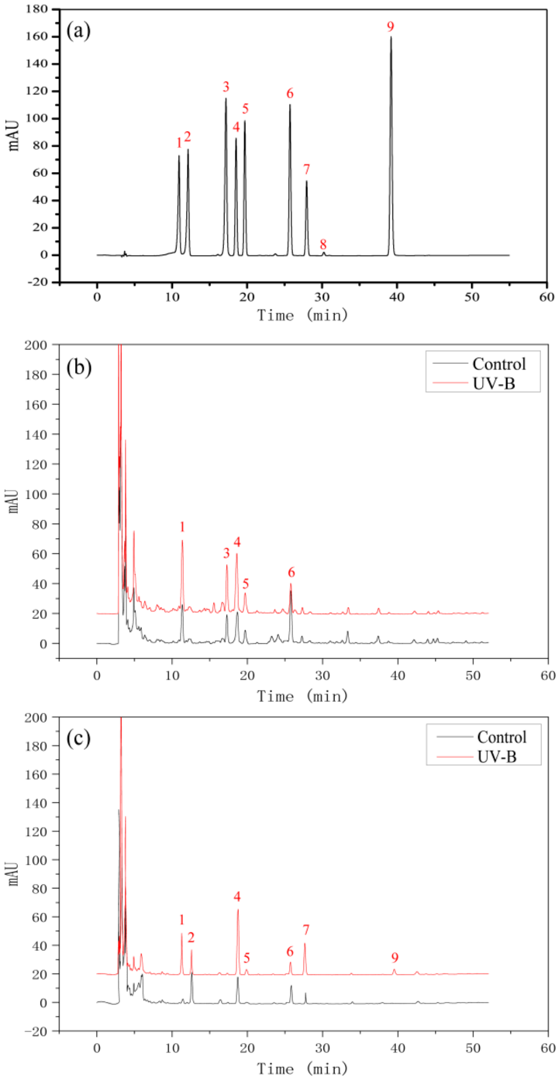

Figure S1. Typical HPLC chromatograms of nine isoflavone monomers in soybean cell suspension cultures. a: Typical HPLC chromatograms of nine isoflavone monomers: 1. daidzin; 2. glycitin; 3. genistin; 4. malonyl daidzin; 5. malonyl glycitin; 6. malonyl genistin; 7. daidzein; 8. glycitein; 9. genistein. b: Typical HPLC chromatograms of isoflavones in soybean hypocotyl cell suspension cultures; c: Typical HPLC chromatograms of isoflavones in soybean cotyledon cell suspension cultures.
